# Outcome of painful bone marrow edema of the femoral head following treatment with parenteral iloprost

**DOI:** 10.4103/0019-5413.45321

**Published:** 2009

**Authors:** Roland Meizer, Dominik Meraner, Elisabeth Meizer, Christian Radda, Franz Landsiedl, Nicolas Aigner

**Affiliations:** Orthopaedic Hospital Vienna-Speising, Speisinger Strasse 109 Vienna, Austria

**Keywords:** Avascular necrosis, bone marrow edema, iloprost, vasoactive drug

## Abstract

**Background:**

Bone marrow edema (BME) is a common cause of hip pain. The aim of the study was to assess the efficacy of the vasoactive drug iloprost in the treatment of BME of femoral head.

**Materials and Methods:**

We reviewed 27 patients (19 male, 8 female) with BME of the femoral head. Their mean age was 53.7 ± 10.8 years. All patients were treated with iloprost, a vasoactive drug that dilates arterioles and venules, reduces capillary permeability and suppresses platelet aggregation. The therapy comprised a series of five infusions with 20 to 50 μg iloprost over 6 h on 5 consecutive days each. Weight bearing was reduced for up to 3 weeks, depending on the severity of symptoms. Pain at rest as well as under stress was assessed with a semi quantitative scale from before and 4 months after therapy. MRI investigations were done before and repeated 4 months after therapy.

**Results:**

At the clinical follow up of four months after therapy, the pain level at rest had diminished by a mean of 58.3% (*P* < 0.0001). Pain under stress decreased by a mean of 41.9% (*P* < 0.0001). On MRI, 20 patients had a significant reduction of BME size or complete normalization and 4 showed no change. Worsening of the MRI pattern was found in 3 patients.

**Conclusion:**

The authors conclude that the use of parenteral iloprost might be a viable method in the treatment of BME of femoral head.

## INTRODUCTION

Bone marrow edema syndrome (BMES) of the hip is being discussed as a possible early reversible stage of avascular necrosis,^1^–^4^ but the incidence of progression from bone marrow edema syndrome to avascular necrosis is still unclear. Other authors consider it to be a distinct, self-limiting, and transient disease^5^,^6^ or a kind of reflex sympathetic dystrophy.^7^,^8^ Although various vascular factors are known to contribute to BMES, the exact pathogenetic processes still remain unknown.^9^,^10^ Injuries to blood vessels caused by disturbances of arteriolar inflow or venous outflow, e.g., by thromboembolism or decreased fibrinolysis,^11^,^12^ have been discussed in this respect. In addition, BMES occasionally occurs in pregnancy.^13^ MRI is the golden standard in diagnosis of BMES. Especially in the T2-weighted fat suppressed STIR sequences, the pathologic changes in the bone marrow can be detected early. Furthermore, differential diagnosis between BMES and other diseases associated with bone marrow edema is possible, as demarcation of osteonecrotic bone areas as well as osteoarthritic changes, bone tumors, osteomyelitis or fractures can clearly be detected with MRI. Bone scans show an increased uptake of radionuclides in bone marrow, but differential diagnosis is not possible with this method. Native radiographs and CT sometimes show focal radiolucent areas after some weeks, reflecting a reduced trabecular mineral content but both methods are neither sensitive nor specific in diagnosing BMES.

When waiting for spontaneous remission, the natural time for improvement of clinical symptoms and normalization in magnetic resonance imaging (MRI) takes from 3 to 18 months.^9^,^14^,^15^ All current treatment options have to be considered as symptomatic therapy since a causative influence on the pathogenetic vascular disturbance has not yet been reported. Because of the reversibility of the disease, nonoperative treatment has been recommended by some authors,^4^,^16^ including reduction of weight bearing, analgesic and anti-inflammatory medication and physiotherapy. The standard surgical treatment is core decompression.^1^,^2^ The aim of our study was to assess the effect of the pharmacological agent iloprost (Ilomedin®, Bayer-Schering AG, Germany), with a spectrum of activity covering all parts of the terminal vascular bed as well as platelet aggregation. The substance has an impact on the rheological properties of the terminal vascular bed. Similar therapeutic effects of iloprost are postulated for bone, where a sinusoidal system of the terminal vascular bed causes a different anatomical situation.

Since 1998, parenterally administered iloprost has been used to treat BMES and early stages of osteonecrosis in our institution.^17^–^19^ It induces vasodilatation and reduction of capillary permeability and inhibits platelet aggregation.^20^ Apart from this rheological efficacy on the terminal vascular bed, it diminishes the concentration of free oxygen radicals^21^ and leukotrienes.^22^–^25^ In clinical use, the most frequent side effects of this substance are headache, nausea and flushes.^20^ Administration is contraindicated in pregnancy, in patients anticoagulated with warfarin or heparin and in cases of concomitant peptic ulcers, heart failure, recent myocardial infarction or unstable angina pectoris.^20^ This approach has been adopted in our hospital and has been giving excellent results in cases of isolated bone marrow edema syndrome. Thus, in contrast to symptomatic treatment, this is the first therapy addressing the vascular abnormalities that are discussed as probable causes for BMES.

The aim of this retrospective study is to assess the efficacy of iloprost concerning reduction of pain and size of BME in the treatment of BME of different etiologies, located in the hip joint.

## MATERIALS AND METHODS

The study was approved by the appropriate ethics committee and was therefore performed in accordance with the ethical standards laid down in the Declaration of Helsinki. The patients between 16-80years with pain in the hip joint and MRI verified BME were included in the study. For all patients, trauma or corticosteroid medication constituted exclusion criteria because of the enhanced risk of osteonecrosis.

We reviewed 27 Caucasian patients (19 male, 8 female) with MRI-verified BME of the femoral head treated between January 2001 and January 2002. Their mean age was 53.7 ± 10.8 years. The mean duration of time from first symptoms to treatment with iloprost was 14.6 weeks. Bone marrow edema was classified as idiopathic in 16 patients and as secondary to osteoarthritis in 11 patients. Standard serological parameters showed hepatopathy in three patients and mild hypercholesterolemia and hypertriglyceridemia in eight patients. Two patients suffered from hypothyroidism, in one patient a nodular goiter had been diagnosed, two patients needed medication because of hypertension and one treatment due to diabetes.

Plain radiographs (anteroposterior and lateral views) were obtained before therapy. MRI were performed before and four months after therapy. For all patients except four, the same 1.0 Tesla MR scanner was used. Coronal turbo SE (spin echo) STIR and T1-weighted MR images of the affected hips were acquired. The STIR sequence, parameters were: repetition time (TR), 2500 ms; echo time (TE), 10 ms; and inversion time (TI), 100 ms. For the T1- weighted sequence, parameters were: TR, 498-750 ms; and TE, 10 ms. A 256 × 256 matrix and a slice thickness of 4 mm were used for both sequences. Both MR examinations (baseline and follow-up) of a particular patient were always performed with the same MR scanner.

Two independent orthopedic surgeons reviewed the MRI pictures and evaluated visually the size and intensity of the BME located in the femoral head. The therapeutic scheme was adjusted to that used for Raynaud's disease in accordance with the pathophysiological hypothesis that BME reflects a disturbance of microcirculation. The therapy comprised a series of five infusions with 20 to 50 *μ*g iloprost on 5 consecutive days each. The drug was administered in 500 ml of sodium chloride solution over 6 h using a drop counter. The daily overall dose of 50 *μ*g was reduced to 20 *μ*g, when we observed frequent side effect. It achieves a dramatic reduction in the frequency and severity of side effects. In cases of major subjective side effects (headache, nausea, flushes), the drugs were discontinued but patient was not excluded from the study. Flow rate was adjusted to side effects, but there was no adjustment for body mass. Symptoms were treated with antiemetic or analgesic drugs.

Weight bearing was reduced by crutches for up to 3 weeks, depending on the severity of symptoms. There was no other special mobilization protocol provided. Pain at rest as well as under stress was assessed with a semi quantitative scale from 0 (no pain) to 5 (unbearable pain) before and 4 months after therapy. Pain categories were calculated as mean values (mean ± SD), and their changes at follow-up were analyzed with the Wilcoxon test. MRI investigations were done before and repeated 4 months after therapy. All patients suffered from severe pain during mechanical loading, and 21 of them also had pain at rest or at night for a mean of 14 weeks (range: 6 to 40 weeks).

Two independent orthopedic surgeons reviewed the MRI pictures and evaluated the size and intensity of the BME located in the femoral head. Changes in BME size were recorded semiquantitatively on a scale from −3 (severe deterioration) to +3 (complete recovery).

## RESULTS

The patients were divided into two dosage subgroups: 9 patients received 50 *μ*g iloprost, and 18 patients 20 *μ*g. Side effects during infusion therapy were seen in 9 patients, i.e., 6 cases of mild headache, 2 cases of mild nausea, and 1 case of flushes. These side effects disappeared within 15 min after the end of infusion.

At the clinical follow up, of 4 months after iloprost therapy, pain at rest had diminished from 1.78 ± 1.29 to 0.74 ± 1.07 (58.3%, *P* < 0.0001): 81% of patients reported a reduction in pain, 15% no change and 4% an increase in pain at rest. Pain under stress decreased from 3.19 ± 0.86 to 1.85 ± 1.43 (41.9%, *P* < 0.0001): 63% of patients had less pain during activity, 37% no change from baseline and no patient had an increase of pain level.

On MRI, 20 patients had a significant reduction of BME size or complete normalization and 4 showed no change. Worsening of the MRI pattern was found in 3 patients. These seven patients were estimated as nonresponders to the treatment with iloprost.

With the reduction of daily dose from 50 to 20 *μ*g, the incidence of side effects could be reduced from 75.0% to 15.8%. Despite lowering the daily iloprost dose from 50 *μ*g to 20 *μ*g, there was no significant difference of reduction of pain and improvement of MRI patterns between dosage subgroups.

## DISCUSSION

The therapeutic options for BMES are limited. Nonoperative management, consisting of symptomatic therapy and reduction of weight bearing until the clinical and radiological findings normalize has been recommended.^4^,^9^,^16^ Although this may be a successful treatment, it may develop into a protracted and incapacitating procedure.^14^ This is underlined by the long duration of symptoms in some of the study patients who had been treated conservatively prior to the study treatment. On the other hand, core decompression gave good results^26^,^27^ based on the theory that elevated intramedullary pressure is the main cause of pain. This method has been recommended for immediate and complete reduction of symptoms, with a return to normal MRI signal patterns.^1^,^2^ However, six weeks of partial or complete weight bearing are recommended. Radke (2003)^28^ presented a series of 22 hips with core decompression, reporting pain relief within 7 days. Two hips progressed to avascular necrosis of the femoral head. Hofmann^2^ presented 43 hips with BMES after core decompression and obtained freedom from symptom within 2 months, with no perioperative complications. Krause^15^ and Calvo^29^ compared core decompression to symptomatic treatment with analgesics and reduced weight bearing and found that the natural course of the disease was significantly shortened by the surgical procedure. Camp and Colwell^30^ as well as Hopson and Siverhus^31^ referred to a high risk of intra- or postoperative fracture (5%–15%) when using core decompression to treat avascular necrosis of the hip. As a new approach to the treatment of the disease, the use of various drugs has provided only limited effects. With regard to pharmacological treatment, Boos^7^ reported good clinical results with sympathetic nerve blockade with bupivacaine in three patients, but no improvement in pathological MRI signal patterns. Laroche^32^ reported some pain reduction in a small controlled study with nifedipine, and Glueck^33^ presented preliminary data using stanozolol in the treatment of avascular necrosis, but these studies were performed in patients with avascular necrosis only and were not followed up in the literature. Lakhanpal^34^ found no beneficial effect with the use of calcitonin, antituberculosis drugs, prednisone and lumbar sympathectomy with bupivacaine.

In contrast to these symptomatic treatment options, we wanted to address the causative pathogenetic vascular abnormalities using the vasoactive compound iloprost. During treatment with iloprost intravenously, most patients showed immediate improvement of symptoms, which was maintained throughout the observation period. Although the clinical outcome was excellent, the occurrence of temporary side effects, such as nausea, vomiting and headache, was also noted. These beneficial effects of treatment with iloprost in BMES of the femoral head are comparable to those that have been published for the treatment of BMES of the talus,^17^ metatarsal bone^18^ and acetabulum.^19^ Excellent results with very fast recovery have been obtained in all cases of isolated bone marrow edema syndrome lacking signs of necrotic areas.^35^ Contrary to other therapeutic options, additional intervention such as reduced weight bearing was not required in most cases. MRI was used to document the regression of the disease during the observation period. In a recent study comparing core decompression with treatment with parenteral iloprost, Aigner^36^ could show that all patients with bone marrow edema syndrome without demarcation of necrotic areas treated with iloprost yielded the same excellent results with a highly accelerated recovery compared to core decompression. Furthermore, most patients resumed normal daily activities earlier.

The validity of our study is limited by the small number of patients involved and the lacking of a control group. A randomized multicenter study will be necessary in order to reach a final conclusion. Nevertheless, our experience with iloprost shows that parenteral use might be a viable method in the treatment of BMES of different origins. Both the natural course of the disease and the normalization of the signal pattern of the MRI seem to be accelerated. Based on the findings of this study, we can recommend iloprost for the nonoperative treatment BME of the femoral head.
